# National Dental Association

**Published:** 1898-10

**Authors:** 


### National Dental Association.

The annual meeting of this body was held at Omaha August 30, 1898. The occasion was one of much interest, partly from the fact that this was the first meeting after the organization. This being a point farther west than the American Dental Association was ever held, it was a question with many whether the attendance would not be greatly lessened, in consequence of that fact this was true to some extent, but there was a very good attendance all things considered.

The profession was represented from nearly all parts of the country, though not as many from some sections as would have been present if the distance had not been so great. A large percent of the representative men were present. Of course, there was quite an amount of routine business in order to put all the machinery into active operation. This consumed more time than is usually given to the work by the predecessor of this body. Nevertheless its scientific work will make a good showing when it comes to be published. The work was highly appreciated by the members.

The indications are that for the next meeting, and the future meetings, commendable progress will be made. We feel warranted in making this statement from the enthusiasm manifested by many of the members, as well as the selection of the next place of meeting.

Dr. Burkhardt, of Batavia, N. Y., was chosen president of the next meeting. This was a most excellent choice and comes as near insuring an interesting meeting for next year as it is possible for one man, and, in addition, he has the aid and co-operation of first-class vice-presidents who will second and support his efforts for a good meeting in 1899. The other officers are tried men, and need no commendation here.

The place chosen (Niagara Falls) is, perhaps, all things considered, the best meeting place in the country for this body. It is, perhaps, as near the center of the dental population of the country as could have been selected. It is an attractive place, it has all needed accommodation and one to which everybody is pleased to go whenever an opportunity affords, and we would suggest that every member and friend of the Association put forth special effort during the coming year to make this one of the greatest meetings of the profession that has ever been held upon the continent.

